# Human Herpes Virus 8 (HHV-8)-Associated Multicentric Castleman Disease in an HIV Patient With Severe Immunosuppression: A Diagnostic Challenge

**DOI:** 10.7759/cureus.111340

**Published:** 2026-06-23

**Authors:** Edmund Birkhamshaw, Ali Abbas, Pyae Paing, Anu Pillai, Akhil Jerry, Kawanpreet Kaur, Farhana Chowdhury

**Affiliations:** 1 Infectious Diseases, Queen Elizabeth Hospital Birmingham, Birmingham, GBR; 2 Internal Medicine, Queen Elizabeth Hospital Birmingham, Birmingham, GBR; 3 Respiratory Medicine, Airedale General Hospital, Keighley, GBR

**Keywords:** febrile illness in hiv patient with low cd4 count, hiv-associated lymphoma, hiv-associated malignancies, human herpes virus 8 (hhv-8) infection, lymphadenopathy in hiv patient, multicentric castleman disease (mcd), rare lymphoproliferative disorders

## Abstract

Human herpes virus 8 (HHV-8)-associated multicentric Castleman disease (HHV-8-MCD) is a rare lymphoproliferative disorder that predominantly affects individuals with advanced immunosuppression, especially people living with HIV. The clinical picture frequently overlaps with opportunistic infections, lymphoma, and haemophagocytic lymphohistiocytosis, creating significant diagnostic challenges.

We present a case of a 51-year-old man recently diagnosed with HIV, who developed recurrent pyrexia, profound hyperferritinaemia, cytopenias, rapidly progressive lymphadenopathy, and escalating HHV-8 viraemia. Lymph node biopsy confirmed the diagnosis of HHV-8-positive MCD. Prompt initiation of rituximab and etoposide resulted in complete clinical and radiological remission.

The case highlights the importance of rapid recognition, timely histological confirmation, early initiation of rituximab-based therapy, and multidisciplinary management in HHV-8-MCD.

## Introduction

Human immunodeficiency virus (HIV) weakens the immune system of affected individuals by impairing cell-mediated immunity (CD4+ T cells). This predisposes to increased risk of malignancy and opportunistic infections. Clinical data suggest a 60-200-fold increased risk of lymphoproliferative disorders, including lymphomas, most notably Hodgkin lymphoma. Additionally, patients are at risk of a rare but severe type of lymphoproliferative disorder called multicentric Castleman disease (MCD), commonly associated with human herpes virus 8 (HHV-8) [1]. 

The pathogenesis of HHV-8-associated multicentric Castleman disease (HHV-8-MCD) lies in a widespread inflammatory response due to excessive cytokine release, mainly interleukin-6 (IL-6), from HHV-8-infected plasmablasts. [2]. Affected individuals classically present with a relapsing-remitting course of constitutional symptoms, including fever, malaise, cytopenias, generalised lymphadenopathy, hepatosplenomegaly and multi-organ involvement. It is frequently associated with concurrent or subsequent HHV-8-related malignancies, including Kaposi sarcoma and non-Hodgkin lymphoma. 

The clinical presentation overlaps with autoimmune conditions and opportunistic infections, resulting in considerable diagnostic challenges for clinicians [3]. Prompt recognition and treatment are crucial, as the condition carries significant morbidity and mortality. Untreated cases of HHV-8-MCD are associated with poor survival; however, rituximab-based treatment has markedly improved outcomes. A prospective cohort of HIV-positive patients demonstrated a five-year overall survival of 92% and relapse-free survival of 82% following rituximab therapy [4]. Despite treatment, HHV-8-MCD remains a relapsing condition, with a reported five-year progression-free survival of approximately 54%, highlighting the need for early diagnosis and ongoing monitoring [5]. 

We describe a case of HHV-8-MCD in a severely immunocompromised HIV-positive individual whose rapid clinical deterioration shortly after antiretroviral initiation posed significant diagnostic complexity and challenges in timely management. 

## Case presentation

A 51-year-old man, a steel fabricator by profession, originally from Zimbabwe and residing in the UK with his family since August 2023, presented to primary care in October 2023 with fatigue, night sweats, and unintentional weight loss that had been ongoing for a few weeks. Clinical examination revealed small-volume lymphadenopathy, with no other abnormal physical findings. Routine investigations arranged by his general practitioner confirmed HIV-1 infection in February 2024, with a baseline CD4 count of 36 cells/µL and an HIV viral load of 168,420 copies/mL. He was referred to HIV outpatient services at this point. 

During the initial consultation in the HIV clinic, he had standard baseline investigations for tuberculosis and other opportunistic infections. He was commenced on antiretroviral therapy with Biktarvy (bictegravir/tenofovir alafenamide/emtricitabine) alongside co-trimoxazole prophylaxis. 

Over the following weeks, he demonstrated clinical improvement, with reduced night sweats and stabilisation of appetite. He remained compliant with highly active antiretroviral treatment, and by mid-March, his HIV viral load had decreased to 70 copies/mL, although CD4 recovery was minimal at that stage. Baseline HHV-8 viral load was 70,000 copies/mL; however, he had no systemic symptoms, no significant lymphadenopathy, or organomegaly at this stage to raise concerns and therefore was managed under close observation. 

On 26 March 2024, during a routine clinic consultation, he reported a six-day history of recurrent fevers (38.5-40 °C) with chills, fatigue, pleuritic left-sided chest discomfort, and new cervical lymph node enlargement. On clinical assessment, he was febrile (39.4°C) and had prominent cervical, supraclavicular, and axillary lymphadenopathy, which progressed over 48 hours. No cutaneous or mucosal Kaposi sarcoma lesions were seen. His chest examination revealed scattered basal crackles without hypoxia. He also had palpable Liver and Spleen. 

His laboratory investigations now demonstrated rising inflammatory markers, including a CRP increase from 72 to 213 mg/L and markedly elevated ferritin at 7,829 ng/mL, with hypoalbuminaemia (albumin 17 g/L). He developed cytopenias, with thrombocytopenia (platelets 89 ×10⁹/L), anaemia (haemoglobin 79 g/L), and neutropenia (neutrophils 1.8 ×10⁹/L). Triglycerides were elevated at 7.3 mmol/L, and lactate dehydrogenase ranged from 329 to 448 U/L. Notably, his HHV-8 viral load increased dramatically to 2.4 million copies/mL. Soluble CD25 was elevated, as was fibrinogen, measured at 6.03 g/L (Table 1). 

**Table 1:** Clinical parameters compared around admission and after treatment completion for HHV-8 MCD HHV-8: Human herpes virus 8; MCD: multicentric Castleman disease

Clinical Parameters	Pre-treatment Values Around Admission	Interpretation	Post-treatment Values	Reference Range
C-reactive protein (CRP)	72 → 213 mg/L	Markedly elevated	4	< 5 mg/L
Ferritin	7,829 ng/mL	Extremely elevated	1877	30–400 ng/mL
Albumin	17 g/L	Hypoalbuminaemia	36	35–50 g/L
Haemoglobin	79 g/L	Anaemia	102	130–170 g/L
Platelets	89 ×10⁹/L	Thrombocytopenia	237 ×10⁹/L	150–400 ×10⁹/L
Neutrophils	1.8 ×10⁹/L	Mild neutropenia	1.67 ×10⁹/L	2.0–7.5 ×10⁹/L
Triglycerides	7.3 mmol/L	Markedly elevated	2.4	< 1.7 mmol/L
Lactate dehydrogenase (LDH)	329–448 U/L	Elevated	264	140–280 U/L
Creatinine	113	elevated	81	64-104 micromol/L
HHV-8 viral load	2.4 ×10⁶ copies/mL	Markedly elevated	Undetectable	Undetectable
HIV viral load	70 copies /ml	Elevated	< 20 copies/ml	<20 copies/ml
Soluble CD25 (sIL-2R)	Elevated	Elevated	Not checked	Low/undetectable
Fibrinogen	6.03 g/L	Elevated	Not checked	2.0–4.0 g/L

Given the combination of escalating HHV-8 viraemia, systemic inflammation, cytopenias, and florid lymphadenopathy, HHV-8-MCD was strongly suspected; however, lymphoma and haemophagocytic lymphohistiocytosis (HLH) remained important differential diagnoses. Due to the risk of potential deterioration and the need for early diagnosis and treatment initiation, he was admitted for inpatient diagnostic workup. 

Extensive infectious screening was done to rule out opportunistic and overlapping infections including CMV, EBV, Toxoplasma, TB, and fungal infections. One of four sputum samples was positive for Mycobacteria chimera, which was felt to be purely asymptomatic colonisation, given the absence of radiological or clinical disease. 

On 28th March, the patient underwent ultrasound-guided biopsy of accessible lymph nodes to secure a diagnosis. Ultrasound revealed multiple abnormal right axillary lymph nodes with loss of fatty hilum, eccentric cortical thickening, and areas of necrosis suggested by cortical heterogeneity, the largest measuring 16 mm in short axis. Core biopsies were obtained to help differentiate between lymphoma, tuberculosis, Kaposi sarcoma, and HHV-8-MCD. 

The patient met four out of five diagnostic criteria for HLH, including fever, splenomegaly, hyperferritinemia, hypertriglyceridemia, and borderline cytopenias. Given this, a bone marrow biopsy was performed to evaluate for HLH. Histological examination of the bone marrow showed no evidence of haemophagocytosis. 

**Figure 1:**
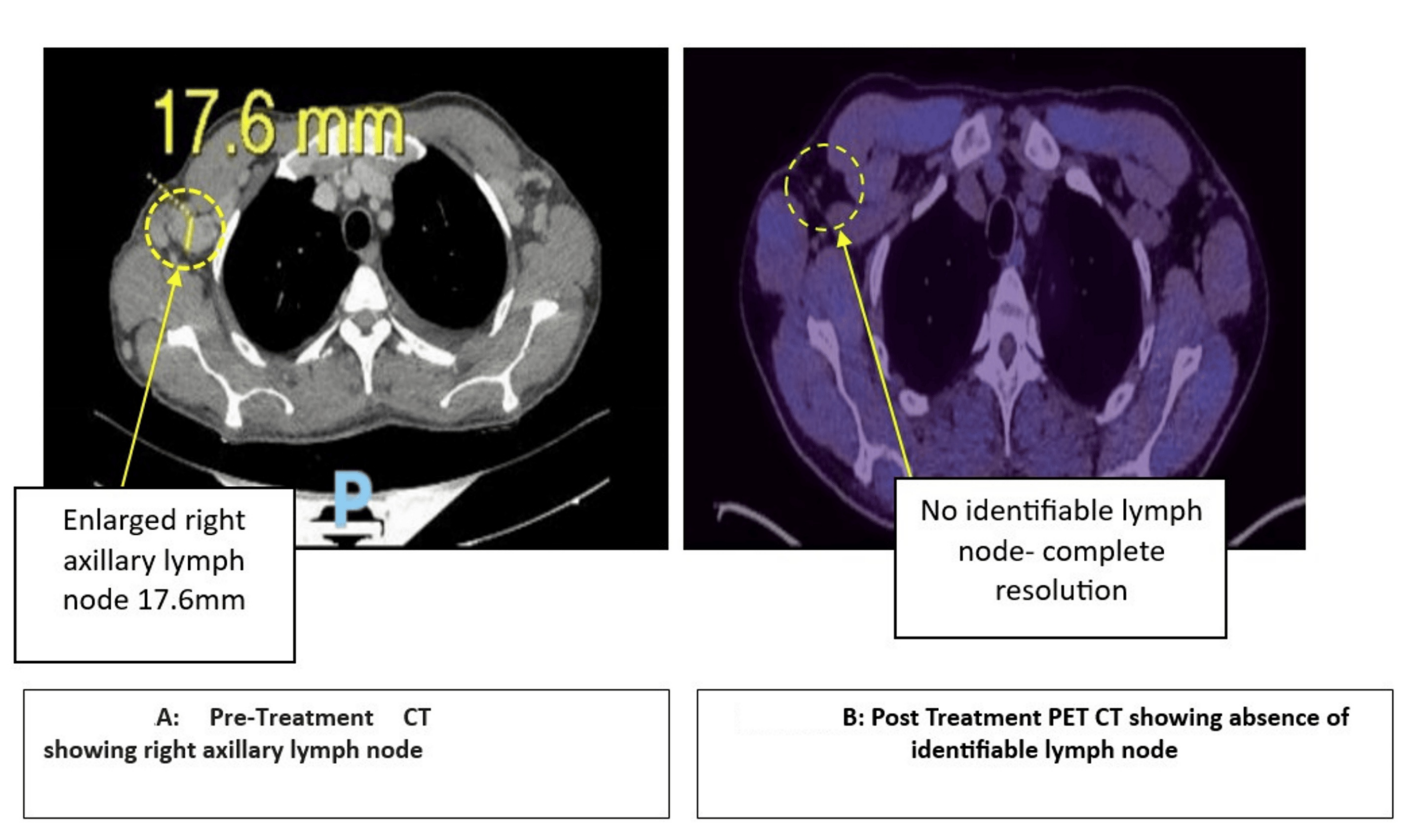
Comparative images of axillary lymph node pre- and post-treatment

**Figure 2:**
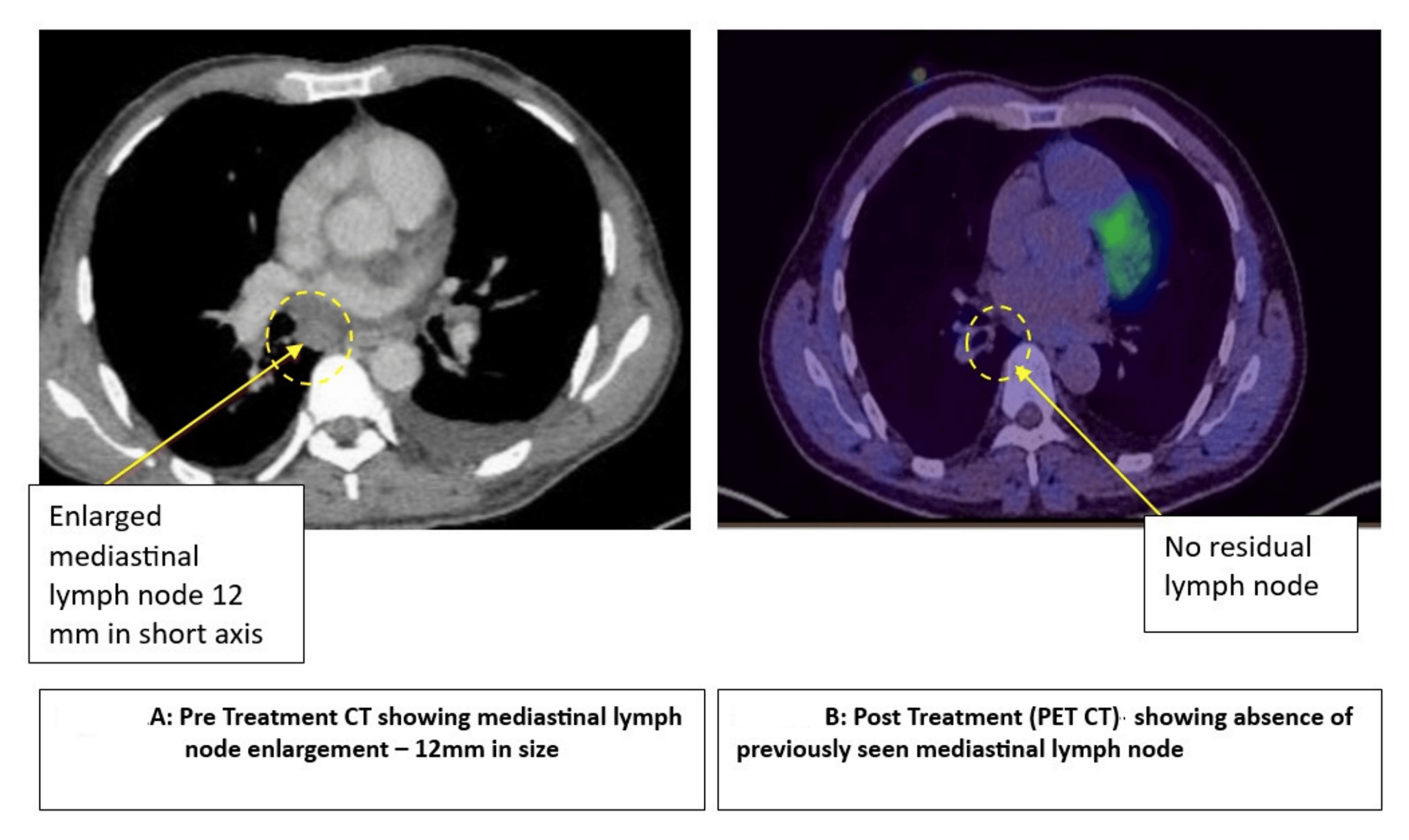
Comparative images of mediastinal lymph nodes pre- and post-treatment

CT from 29th March demonstrated widespread lymphadenopathy involving cervical, supraclavicular, mediastinal, axillary, and abdominal regions, with the largest right axillary lymph node measuring 18 mm in short axis. Mediastinal lymph nodes were enlarged compared to the February 2024 HRCT. Hepatosplenomegaly was noted, with the spleen measuring 14.7 cm. Small-volume bilateral inguinal lymph nodes were present (largest left 9 mm), and large but elongated right external iliac lymph nodes measured 12.2 mm. His HRCT from the previous month had shown small-volume mediastinal and axillary lymph node enlargement, but there was no bulky nodal disease at that time (Figures 1, 2). 

**Figure 3:**
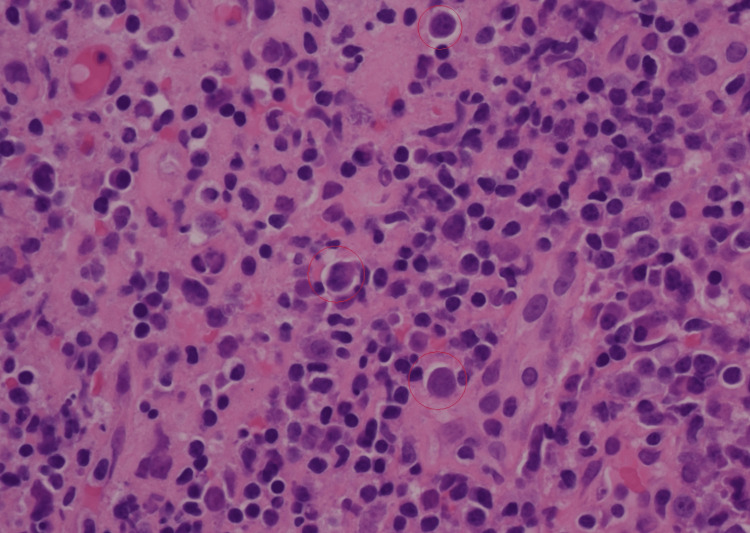
Right axillary lymph node core biopsy - H & E staining showing plasmablasts and diffuse plasma cells proliferation - classical for HHV-8 multicentric Castleman syndrome (red circle) Image received from Histopathology Lab, Queen Elizabeth Hospital Birmingham, UK HHV-8: Human herpes virus 8

**Figure 4:**
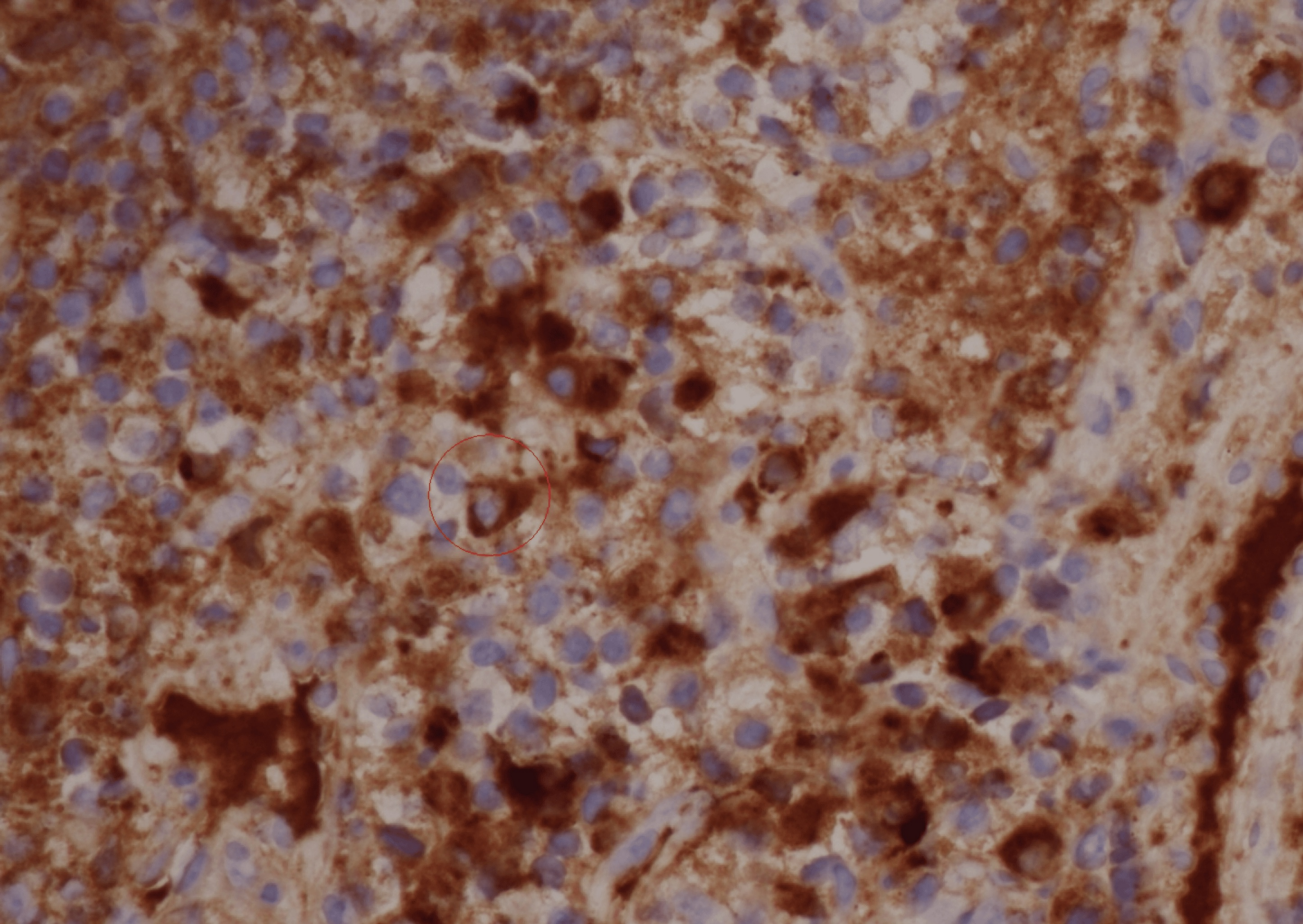
Axillary lymph node histology with HHV-8 LANA (latency-associated nuclear antigen) immunohistochemistry demonstrating positive HHV-8-infected plasmablasts with large nuclei (red circle) Image received from Histopathology Lab, Queen Elizabeth Hospital Birmingham, UK HHV-8: Human herpes virus 8

The diagnosis was confirmed with histology from the axillary lymph node demonstrating latency-associated nuclear antigen (LANA-1)-positive plasmablasts characteristic of HHV-8 infection, polytypic plasma cells, and no evidence of Kaposi sarcoma. These findings were consistent with HHV-8-positive MCD (Figures 3, 4) [6]. 

Following diagnostic confirmation of HHV-8-MCD, treatment with rituximab (375 mg/m² weekly) and etoposide (100 mg/m² weekly) was initiated. Antiretroviral therapy and co-trimoxazole were continued, and valacyclovir prophylaxis for herpes and varicella zoster virus (HZV/VZV) was added. The patient completed four cycles of combined therapy in total, with the last two delivered as an outpatient.

His clinical progress was reviewed in the haematology clinic following completion of the treatment. His fever resolved promptly, lymphadenopathy regressed markedly, and inflammatory markers and cytopenias normalised. Follow-up PET imaging demonstrated complete metabolic remission with no residual FDG-avid disease detected (Figures 1B, 2B). 

## Discussion

HHV-8-MCD remains an important differential diagnosis in patients with advanced HIV infection presenting with fever, lymphadenopathy, cytopenias, and systemic inflammation. Diagnosis is often challenging because of its clinical overlap with opportunistic infections, lymphoma, immune reconstitution inflammatory syndrome (IRIS), and HLH.

HHV-8-MCD is characterised by cytokine-driven systemic inflammation resulting from HHV-8-infected plasmablasts producing viral IL-6. Previous studies have described a relatively consistent clinical phenotype comprising fever, weight loss, diffuse lymphadenopathy, hepatosplenomegaly, and inflammatory cytopenias [7,8]. Histological confirmation through lymph node biopsy with HHV-8 LANA-1 staining remains the diagnostic gold standard, highlighting the importance of early tissue sampling when the diagnosis is suspected.

Our case offers several important clinical and educational insights. First, the patient developed rapidly progressive systemic inflammation shortly after initiation of effective antiretroviral therapy, creating a complex diagnostic scenario in which HHV-8-MCD, opportunistic infection, lymphoma, HLH, and IRIS all needed to be considered. This temporal relationship increased diagnostic uncertainty and highlights the need to maintain a high index of suspicion for HHV-8-MCD, even in patients demonstrating an excellent virological response to antiretroviral therapy.

Second, despite marked systemic inflammation, the burden of lymphadenopathy was relatively modest, with the largest lymph node measuring only 18 mm in short axis. This illustrates that severe HHV-8-MCD activity can occur in the absence of bulky nodal disease and that relatively small lymph nodes should not reassure clinicians when other features suggest an aggressive inflammatory process.

Third, the patient demonstrated a striking rise in HHV-8 viraemia, from 70,000 copies/mL at the time of HIV diagnosis to 2.4 million copies/mL in parallel with clinical deterioration. Following treatment, the viral load became undetectable. This close correlation between HHV-8 viral load kinetics and disease activity highlights the potential value of serial HHV-8 viral load monitoring in identifying evolving HHV-8-MCD and assessing treatment response in patients with advanced HIV infection.

Fourth, the patient fulfilled several diagnostic criteria for HLH, including fever, splenomegaly, hyperferritinaemia, hypertriglyceridaemia, and cytopenias, yet bone marrow examination demonstrated no evidence of haemophagocytosis. This case highlights the significant diagnostic overlap between HHV-8-MCD and HLH and demonstrates how HHV-8-MCD can closely mimic HLH without meeting criteria for overt haemophagocytic syndrome.

Finally, despite several adverse prognostic features, including profound immunosuppression (CD4 count 36 cells/µL), marked inflammatory activation, organomegaly, and extreme HHV-8 viraemia, the patient achieved complete metabolic remission following prompt diagnosis and initiation of rituximab-etoposide therapy. This favourable outcome reinforces the importance of early recognition, expedited tissue diagnosis, and multidisciplinary management in severe HHV-8-associated MCD.

The principal contribution of this case is therefore not the description of a single novel clinical feature, but rather the illustration of how rapidly evolving HHV-8-MCD can present in advanced HIV infection with overlapping features of IRIS, HLH, lymphoma, and opportunistic infection. It also highlights the potential value of serial HHV-8 viral load monitoring and early lymph node biopsy in facilitating timely diagnosis and treatment, even in patients with apparently limited nodal disease.

## Conclusions

This case highlights how rapidly progressive HHV-8-MCD can develop despite an initial virological response to antiretroviral therapy and demonstrates the potential value of serial HHV-8 viral load monitoring in identifying evolving disease activity. It also illustrates that severe inflammatory disease may occur despite relatively limited lymphadenopathy, reinforcing the importance of maintaining clinical suspicion even in the absence of bulky nodal disease.

Early tissue diagnosis with HHV-8-specific histopathological staining, together with prompt multidisciplinary management and rituximab-based therapy, can result in excellent outcomes even in patients with profound immunosuppression and other high-risk features.

## References

[1] Naif HM (2013). Pathogenesis of HIV infection. Infect Dis Rep.

[2] Dispenzieri A, Fajgenbaum DC (2020). Overview of Castleman disease. Blood.

[3] Fajgenbaum DC, Uldrick TS, Bagg A (2017). International, evidence-based consensus diagnostic criteria for HHV-8-negative/idiopathic multicentric Castleman disease. Blood.

[4] Gérard L, Michot JM, Burcheri S (2012). Rituximab decreases the risk of lymphoma in patients with HIV-associated multicentric Castleman disease. Blood.

[5] Liu W, Cai Q, Yu T (2022). Clinical characteristics and outcomes of Castleman disease: a multicenter Consortium study of 428 patients with 15-year follow-up. Am J Cancer Res.

[6] Dupin N, Diss TL, Kellam P (2000). HHV-8 is associated with a plasmablastic variant of Castleman disease that is linked to HHV-8-positive plasmablastic lymphoma. Blood.

[7] Bower M, Powles T, Williams S (2007). Brief communication: rituximab in HIV-associated multicentric Castleman disease. Ann Intern Med.

[8] Chan KL, Lade S, Prince HM, Harrison SJ (2016). Update and new approaches in the treatment of Castleman disease. J Blood Med.

